# In This Issue

**Published:** 1998

## ALCOHOL USE AMONG ADOLESCENTS

Although alcohol consumption is illegal for people younger than age 21, adolescents continue to drink alcohol. Drs. Patrick M. O’Malley, Lloyd D. Johnston, and Jerald G. Bachman review the findings of three ongoing national surveillance systems that assess alcohol use among adolescents. The surveys indicate widespread alcohol use among youth; indeed, up to 54 percent of 8th graders and up to 84 percent of 12th graders report having consumed alcohol. The authors discuss these findings along with data showing that the prevalence of both drinking and getting drunk increases with age. Even more disturbing is that adolescent alcohol use and its associated consequences, including heavy drinking and driving after drinking, have increased in recent years, despite an earlier period of decline. (pp. 85–93)

## DIAGNOSIS AND ASSESSMENT OF ALCOHOL USE DISORDERS

Correct diagnosis of an illness, including alcohol abuse and alcoholism, hinges on the validity of the diagnostic system used. An accurate diagnostic system helps physicians determine the course, prognosis, and treatment of a disorder and aids researchers in understanding the mechanisms underlying the condition. The criteria used for diagnosing alcohol problems in adolescents are based on guidelines first established for adults. According to Drs. Christopher S. Martin and Ken C. Winters, however, some of the symptoms assessed with the current diagnostic systems may not typically appear in adolescent drinkers, and the criteria may not delineate the different levels of drinking problems that exist in adolescents. Despite those limitations, assessment tools based on adult diagnostic criteria offer valuable clues for clinicians and researchers who deal with alcohol problems in adolescents. (pp. 95–105)

## LESSONS FROM PROJECT NORTHLAND

Project Northland is an ongoing community trial aimed at reducing alcohol use and alcohol-related problems among adolescents. The project combines strategies to encourage youth not to drink with strategies to both reduce the availability of alcohol and modify society’s attitudes toward youthful drinking. Drs. Carolyn L. Williams and Cheryl L. Perry review the strategies behind Project Northland, a project that is following a group of adolescents from sixth grade to their high school graduation in 1999. Project Northland has incorporated a number of promising prevention strategies that target specific aspects of adolescents’ social environment. The authors review the project’s early results and discuss the special challenges associated with preventing older adolescents from obtaining and using alcohol. (pp. 107–116)

## PSYCHOPATHOLOGY IN ADOLESCENT ALCOHOL ABUSE AND DEPENDENCE

Adolescents who abuse or are dependent on alcohol often have coexisting mental conditions. For example, those children often are antisocial and may experience bouts of depression and anxiety. Those disorders may exist well before alcohol use begins, or they may be a result of drinking. Drs. Duncan B. Clark and Oscar G. Bukstein examine the link between alcohol use disorders and some common mental disorders. The authors discuss the value of assessing adolescents for alcohol use disorders as well as other mental disorders. Drs. Clark and Bukstein stress that multiple treatment approaches may be necessary in adolescents who have multiple types of disorders. (pp. 117–126)

## AOD USE AND ATTENTION DEFICIT/HYPERACTIVITY DISORDER

Children with attention deficit/ hyperactivity disorder (ADHD) have difficulty focusing on common academic, work-related, and social tasks. This impairment continues throughout adulthood. ADHD also has been found to be an important risk factor for the development of alcohol and other drug use disorders (AODD). According to Dr. Timothy E. Wilens, people with ADHD who engage in AOD abuse are less likely to achieve abstinence or to moderate their AOD use than are those without ADHD, especially when other psychiatric disorders are present. Dr. Wilens explores the relationship between ADHD and addictive disorders and discusses treatment approaches for ADHD that might forestall the subsequent development of AODD. The identification of specific risk factors for AOD abuse in people with ADHD may permit more targeted prevention and treatment programs for both disorders. (pp. 127–130)

## A DEVELOPMENTAL BEHAVIOR-GENETIC PERSPECTIVE ON ALCOHOLISM RISK

The symptoms of alcoholism typically do not become evident until a person reaches adulthood. Yet the warning signs of an increased risk for developing alcoholism may be apparent in early childhood. According to Dr. Richard J. Rose, children as young as age 3 may exhibit certain characteristics, such as impatience and aggressiveness, that can indicate an increased risk for alcoholism. Studies of twins who were raised apart show that both genetic and environmental factors are at work. For example, environmental factors, such as an older sibling’s influence, are the key reason that a youngster begins to drink alcohol. Genetic factors, evidenced by a family history of alcoholism, help determine the frequency of drinking once alcohol use has begun. (pp. 131–143)

## THE IMPACT OF A FAMILY HISTORY OF ALCOHOLISM ON THE RELATIONSHIP BETWEEN AGE AT ONSET OF ALCOHOL USE AND DSM–IV ALCOHOL DEPENDENCE

Among the factors that contribute to a person’s risk for alcohol dependence are the age at which alcohol use first occurs and the person’s family history of alcoholism. In NIAAA’s Epidemiologic Bulletin No. 39, Dr. Bridget F. Grant explores the relationship between age at onset of alcohol use, family history of alcoholism, and lifetime prevalence of alcohol dependence, based on data obtained in the National Longitudinal Alcohol Epidemiologic Survey. Those analyses indicate that regardless of gender, race, or family history of alcoholism, the earlier a person begins drinking, the more likely he or she is to become dependent on alcohol. Moreover, in all the population subgroups studied, and regardless of the age of initiation of alcohol use, the prevalence of lifetime alcohol dependence is greater in people with a family history of alcoholism than in people without such a history. (pp. 144–148)

